# Artesunate relieves acute kidney injury through inhibiting macrophagic Mincle‐mediated necroptosis and inflammation to tubular epithelial cell

**DOI:** 10.1111/jcmm.16833

**Published:** 2021-08-01

**Authors:** Xian‐ying Lei, Rui‐zhi Tan, Jian Jia, Song‐lin Wu, Cheng‐li Wen, Xiao Lin, Huan Wang, Zhang‐jing Shi, Bo Li, Yan Kang, Li Wang

**Affiliations:** ^1^ Department of Critical Care Medicine West China Hospital Sichuan University Chengdu China; ^2^ ICU The Affiliated Hospital of Southwest Medical University Luzhou China; ^3^ Research Center of Integrated Traditional Chinese and Western Medicine Affiliated Traditional Medicine Hospital Southwest Medical University Luzhou China

**Keywords:** AKI, artesunate, inflammation, Mincle, necroptosis

## Abstract

Artesunate is a widely used derivative of artemisinin for malaria. Recent researches have shown that artesunate has a significant anti‐inflammatory effect on many diseases. However, its effect on acute kidney injury with a significant inflammatory response is not clear. In this study, we established a cisplatin‐induced AKI mouse model and a co‐culture system of BMDM and tubular epithelial cells (mTEC) to verify the renoprotective and anti‐inflammatory effects of artesunate on AKI, and explored the underlying mechanism. We found that artesunate strongly down‐regulated the serum creatinine and BUN levels in AKI mice, reduced the necroptosis of tubular cells and down‐regulated the expression of the tubular injury molecule Tim‐1. On the other hand, artesunate strongly inhibited the mRNA expression of inflammatory cytokines (IL‐1β, IL‐6 and TNF‐α), protein levels of inflammatory signals (iNOS and NF‐κB) and necroptosis signals (RIPK1, RIPK3 and MLKL) in kidney of AKI mouse. Notably, the co‐culture system proved that Mincle in macrophage can aggravate the inflammation and necroptosis of mTEC induced by LPS, and artesunate suppressed the expression of Mincle in macrophage of kidney in AKI mouse. Overexpression of Mincle in BMDM restored the damage and necroptosis inhibited by artesunate in mTEC, indicating Mincle in macrophage is the target of artesunate to protect tubule cells in AKI. Our findings demonstrated that artesunate can significantly improve renal function in AKI, which may be related to the inhibition of Mincle‐mediated macrophage inflammation, thereby reducing the damage and necroptosis to tubular cells that provide new option for the treatment of AKI.

## 1 INTRODUCTION

Acute kidney injury (AKI) is a complex syndrome with high morbidity and mortality.^1^ Recent study reported that the morbidity of AKI in hospitalized patients is as high as 10%–15%, and in ICU patients even exceeds 50%.^2^ Normally, the major causes and pathophysiological mechanisms for AKI include renal hypoperfusion, cardiorenal syndrome, nephrotoxin exposure, sepsis, major surgery, intra‐abdominal hypertension, rapidly progressive glomerulonephritis and acute interstitial nephritis.^3^ As AKI seriously threatens the health of patients and can significantly increase the risk of CKD, more and more studies have begun to pay attention to the pathogenesis and treatment of AKI. Notably, AKI has been known to be linked to intrarenal and systemic oxidative stress^4^ and inflammation.^5^, ^6^ Accumulating evidence has proved that inflammation plays an important role in the development of AKI, which aggravates the injury of renal tubular epithelial cell and damages the renal function in a rapid time.^7^, ^8^, ^9^ Therefore, exploring the cellular and molecular mechanisms of inflammation in kidney of AKI has an important role for the treatment of AKI. In this process, almost all immune cells are believed to be involved in the inflammation of AKI kidney, especially of dendritic cells, monocytes/macrophages, neutrophils, T lymphocytes and B lymphocytes,^10^ in which macrophage has been proved to play an important role in the process of renal inflammation.^11^, ^12^ The deletion of macrophage in kidney protects renal function from IRI injury.^13^ Therefore, macrophages are an important target cell for research and treatment of inflammation‐induced injury in kidney of AKI.

Macrophage inducible C‐type lectin (Mincle) is a pattern recognition receptor, which can recognize damage‐associated molecular patterns (DAMPs) and pathogen‐associated molecular patterns (PAMPS), and mainly expressed on membrane of monocyte, macrophage and dendritic cell.^14^ At first, Mincle was considered to be an active receptor for a variety of pathogenic bacteria,^15^ even it is currently believed to enhance the response to *Streptococcus pneumoniae* in mice and aggravate inflammation.^16^ However, more and more studies have shown that Mincle is also involved in various other pathological and physiological activities, including promoting fibrosis,^17^ tumour formation^18^ and infection.^19^ A recent study demonstrated that Mincle is essential for maintaining the M1 phenotype of macrophage in kidney of AKI, and down‐regulation of Mincle in macrophage relieved the renal injury, suggesting that Mincle is a key promotor for macrophagic inflammation in AKI.^20^ Our previous research also proved that inhibition of Mincle‐related signal pathway protects kidney from AKI injury.^21^ Therefore, drug intervention targeting Mincle may be the key to the treatment of AKI.

Artemisinins are a class of sesquiterpene trioxane lactone drugs widely used in anti‐malaria.^22^ Artesunate, artemether and arteether are the mainly used derivatives of artemisinin for malaria in worldwide.^23^ Among them, artesunate (ART) is the most studied artemisinin because of its better water solubility and higher oral bioavailability.^24^ In addition to anti‐malarial effect, in recent years, more and more studies have reported that artesunate has significant anti‐inflammatory, antioxidant and anti‐autophagy effects. A recent study demonstrated that the inhibitory effect of artesunate on ulcerative colitis is associated with its suppressing excessive ER stress‐mediated intestinal barrier damage and inflammatory response.^25^ On the other hand, artesunate could be used to treat airway remodelling by regulating PPAR‐γ/TGF‐β1/Smad signalling in the context of chronic obstructive pulmonary disease.^26^ Artesunate can also against hepatic ischaemia/reperfusion‐induced inflammasomopathy by interrupting cross‐talk of inflammatory and oxidative stress trajectories signifies.^27^ In view of the significant anti‐inflammatory effect of artesunate, we hope to explore whether artesunate can be used to against renal inflammation induced by acute kidney injury, as well as its improvement of kidney function and potential mechanisms.

## 2 MATERIALS AND METHODS

### 2.1 Animal experiments

Thirty‐six male C57BL/6 mice with age of 8 weeks and weight of 22–25 g were purchased from Chongqing Tengxin Biotechnology Co., Ltd. and housed in a temperature‐ and humidity‐controlled room with a 12‐h light/12‐h dark cycle. Mice were divided into 6 groups, including ctrl group (Ctrl), cisplatin‐induced AKI group (Cis), 7.5 mg/kg/day artesunate‐treated AKI group (Cis + ART‐L), 15 mg/kg/day artesunate‐treated AKI group (Cis + ART‐H), 15 mg/kg/day artesunate group (ART‐H) and 100 mg/kg/day curcumin treated‐AKI group (Cis + Cur). Curcumin was served as a positive control.^28^ Artesunate and curcumin were gavaged to mice in treatment group 1 h before injection of cisplatin (20 mg/kg), then continuously administrated to mice once a day until kill of all mice on day 3. All animal experiments were carried out according to the guidelines approved by the Animal Ethics Committee of Southwest Medical University (Permit number: 201903‐148).

### 2.2 Isolation of BMDM and mTEC

Bone marrow–derived macrophages (BMDM) were isolated from the tibia and femur of C57BL/6 mice and differentiated in low‐glucose DMEM medium containing 30% supernatant of L929 cell for 7 days following the protocol in our previous study.^21^ To obtain the primary tubular epithelial cells (mTEC) from mouse, the kidney was digested with 3 mg/ml Collagen 4 at 37℃ for 15min, then filtered the suspension on a 70‐μm cell strainer and centrifuged at 120 *g* for 5 min, followed by culturing the tubular epithelial cells in F12/DMEM medium with 5% foetal bovine serum containing 50 ng/ml epidermal growth factor and 5 μl/ml ITS‐G for 5 days. All cells were incubated in a 37℃ incubator containing a constant 5% CO_2_.

### 2.3 Cell culture

The primary BMDM cells were cultured in Dulbecco modified Eagle's medium (DMEM, Sigma‐Aldrich) supplemented with 30% L929 supernatant, 10% foetal bovine serum (Gibco), 100 U/ml penicillin and 100 mg/ml streptomycin at 37℃ with 5% CO_2_. To establish the macrophage inflammatory model, BMDM were incubated with 200 ng/ml lipopolysaccharide (LPS) for protein extraction for 24 h and RNA extraction for 6 h. The primary mTEC cells were cultured in F12/DMEM (Sigma‐Aldrich) supplemented with 5% FBS, 100 U/ml penicillin and 100 mg/ml streptomycin at 37℃ with 5% CO_2_. The concentration of artesunate used in the cellular experiments was 2 and 10 μg/ml.

### 2.4 MTT

A total of 5000 BMDM of mTEC cells were seeded in a well of 96‐well plate. The next day, replace the medium with drug‐containing culture medium for 24 h culture, followed by discarding the supernatant and adding 0.5% MTT‐containing medium for 4 h. After using DMSO to dissolve the purple formazan, we measure the absorbance by a microplate reader at 570 nm with a reference absorbance at 630 nm.

### 2.5 Detection of renal function

Serum creatinine and BUN of each group were detected by renal function detection kits ordered from Jiancheng Bioengineering Institute following the product manual. The acute tubular necrosis score was obtained by evaluating casts, brush border loss, tubular dilation, necrosis and calcification in kidney of each group from H&E staining.^29^

### 2.6 ELISA

Enzyme‐linked immunosorbent assay kits were used to measure the concentration of IL‐1β, IL‐6 and TNF‐α in supernatant of BMDM cells following the instruction of kits (IL‐1β [EMC001B], IL‐6 [EMC004] and TNF‐α [EMC102A], Neobioscience). The concentration of each cytokine was defined by the absorbance measured with a microplate reader at 450 nm.

### 2.7 Pathological staining

After fixing the kidney samples with 4% paraformaldehyde and embedding in paraffin, samples were cut into 4‐μm serial sections. Subsequently, the samples were dewaxed in xylene and rehydrated in ethanol gradients and then stained with Haematoxylin and Eosin (Beyotime) for H&E staining, as well as 1% periodic acid and Periodic Acid–Schiff reaction for PAS staining. Images were captured by a light microscope (Eclipse 80i, Nikon).

### 2.8 Immunohistochemistry

After deparaffinization and rehydration of paraffin sections, the samples were blocked with 5% BSA for 30 min at RT, followed by incubating with primary anti‐F4/80 (1:100, Santa Cruz) antibody at 4℃ overnight. Subsequently, samples were incubated with a secondary antibody (horseradish peroxidase–conjugated goat anti‐rat antibody). Ultimately, the antibody binding was visualized using DAB kit (ZLI‐9017, ZSGB‐BIO). Images were captured by a light microscope (Eclipse 80i, Nikon).

### 2.9 Flow cytometry

Mouse kidney samples were digested with Collagen 4 at 37℃ for 30 min, and the mTEC cells were digested with 0.25% trypsin‐EDTA at 37℃ for 5 min into cell suspension. All cells were fixed with 4% paraformaldehyde for 15 min. Then, the cells from kidney were incubated with primary anti‐Mincle (1:100, Santa Cruz), anti‐F4/80 (1:100, Santa Cruz) and anti‐iNOS (1:100, CST) antibodies at 4℃ overnight. Then, cells were incubated with FITC‐, PE‐ or APC‐conjugated secondary antibodies at RT for 1 h. The mTEC cells were incubated with Annexin V‐FITC and PI staining solution (Vazyme) for 10 min at RT. All the cells were gated and analysed by using a flow cytometer (BD biosciences), and the Flow data were processed by using FlowJo software (V10).

### 2.10 Real‐time PCR

Total RNA was obtained from cells and kidney by using TRIzol reagent. Total RNA of each sample was reverse‐transcribed to cDNA by using Reverse Transcription Kit. The mRNA expression levels of each gene were determined by Mastercycler EP Realplex2 real‐time PCR system. PCR amplification was carried out for 40 cycles. The sequences of each primer are listed in Table 1.

**TABLE 1:** Specific primers for real‐time PCR

Gene	Primer sequence (5′–3′)
Mincle	S: ACCAAATCGCCTGCATCC
	A: CACTTGGGAGTTTTTGAAGCATC
Tim‐1	S: ACATATCGTGGAATCACAACGAC
	A: ACAAGCAGAAGATGGGCATTG
RIPK1	S: GAAGACAGACCTAGACAGCGG
	A: CCAGTAGCTTCACCACTCGAC
RIPK3	S: TCTGTCAAGTTATGGCCTACTGG
	A: GGAACACGACTCCGAACCC
Cyclin D1	S: GCGTACCCTGACACCAATCTC
	A: CTCCTCTTCGCACTTCTGCTC
IL‐1β	S: TGCCACCTTTTGACAGTGATG
	A: AAGGTCCACGGGAAAGACAC
IL‐6	S: AAAGAGTTGTGCAATGGCAATTCT
	A: AAGTGCATCATCGTTGTTCATACA
TNF‐α	S: CATCTTCTCAAAATTCGAGTGACAA
	A: TGGGAGTAGACAAGGTACAACCC
iNOS	S: CAGCTGGGTCGTACAAAC
	A: CATTGGAAGTGAAGCGTTT
GAPDH	S: ACAGCAACAGGGTGGTGGAC
	A: TTTGAGGGTGCAGCGAACTT

### 2.11 Western blot

Proteins from kidney and cells were isolated by using RIPA lysis buffer with PMSF. Briefly, samples treated with RIPA for 30 min on ice and followed by discarding the supernatant after 13,000 *g* centrifugation. Equal amounts of protein lysates were resolved in 10% SDS‐PAGE gel and transferred to a PVDF membranes. Then, the membranes were incubated with primary antibodies against Tim‐1 (1:1000, CST), iNOS (1:1000, CST), Mincle (1:500, Santa Cruz), p‐p65 (1:500, Santa Cruz), p65 (1:500, Santa Cruz), p‐p50 (1:500, Santa Cruz), p50 (1:500, Santa Cruz), p‐Syk (1:1000, CST), Syk (1:1000, CST), p‐MLKL (1:1000, CST), MLKL (1:1000, CST), p‐RIPK1 (1:1000, CST), RIPK1 (1:500, Santa Cruz), p‐RIPK3 (1:1000, CST) and RIPK3 (1:500, Santa Cruz) at 4℃ overnight. Subsequently, membranes were incubated with the corresponding secondary antibodies (peroxidase‐conjugated goat anti‐mouse IgG and goat anti‐rabbit IgG) at RT for 1 h. Images of bands were captured by using a chemiluminescence imaging system (ChemiScope 6200, Clinx), and the grey intensity of the bands was calculated by ImageJ software.

### 2.12 Co‐culture system

BMDM and mTEC cells were co‐cultured by using Millicell^®^ Hanging Cell Culture Inserts system (Millipore). mTEC cells were seeded in the well of 6‐well or 12‐well plates, and the next day, collected BMDM cells were added into the upper compartment (insert) to make physically separation of BMDM and mTEC. Subsequently, co‐culture of these two types of cells with LPS and/or ART for 6 h to isolate RNA and 24 h to extract protein from mTEC, respectively.

### 2.13 Genetic modify of Mincle in BMDM

pcDNA3.1‐Mincle plasmid (GeneChem) and Mincle siRNA (Sangon Biotech, China) were transfected to BMDM by DNA transfection reagent (ZETA life) to construct the Mincle overexpression and knockdown cellular model, respectively. The sequences of Mincle siRNA were as follows: sense: CCUUUGAACUGGAAACAUUTT, and antisense: AAUGUUUCCAGUUCAAAGGTT.

### 2.14 Statistics

Data were presented as the mean ± standard deviation. Data analysis was performed with one‐way analysis of variance test by using SPSS software 21.0 (SPSS); *p* value <0.05 was considered to be statistically significant.

## 3 RESULTS

### 3.1 Artesunate ameliorated renal dysfunction in cisplatin‐induced AKI

**FIGURE 1:**
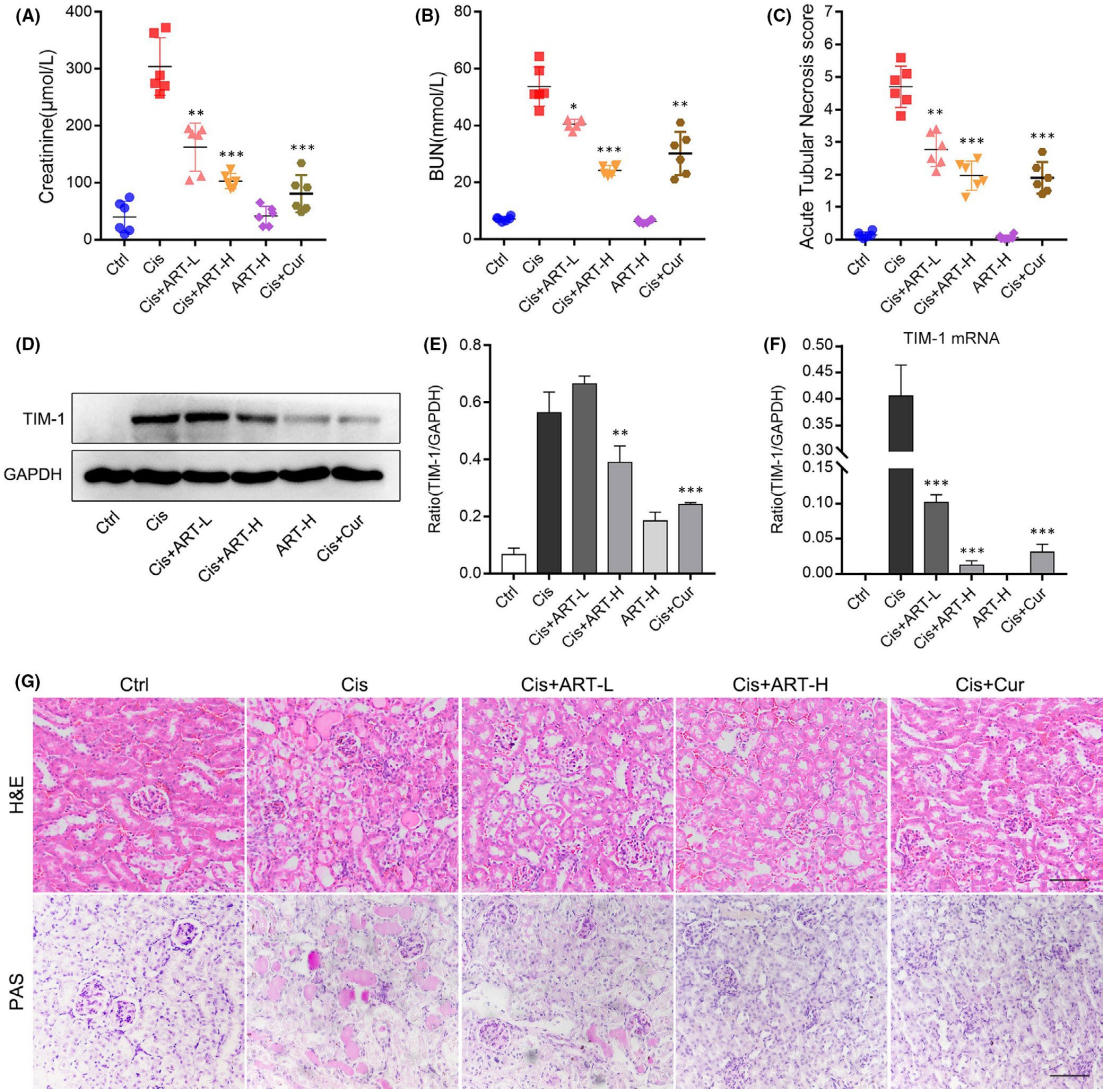
Artesunate ameliorated renal dysfunction in cisplatin‐induced AKI. (A) ART administration effectively reduced the level of serum creatinine (CREA) in AKI mice. (B) ART administration strongly down‐regulated the level of serum urea nitrogen (BUN) in AKI mice. (C) The results of acute tubular necrosis score. (D and E) The protein level of Tim‐1 detected by Western blotting normalized to GAPDH. (F) ART administration significantly reduced the mRNA expression of Tim‐1 in kidney of AKI mice. (G) H&E staining and PAS staining results of kidney of mice in each group. *p < 0.05, **p < 0.01, ***p < 0.001 vs. Cis group

In this study, we established a cisplatin‐induced mouse model of AKI to observe the therapeutic effect of ART on renal dysfunction against cis‐induced AKI in vivo. After 72 h of cisplatin injection, the results showed a significant increase in Scr and BUN levels. Dramatically, the enhanced Scr and BUN levels were strikingly decreased by ART treatment (Figure 1A,B). The results of acute tubular necrosis score indicated that ART can effectively attenuated Cis‐induced renal injury (Figure 1C). Kidney injury molecule‐1 (Kim‐1, also known as Tim‐1) is an emblematic kidney injury biomarker, whose mRNA and protein levels were remarkably reduced by ART in kidney of AKI mouse, suggesting that ART significantly improved renal tubular injury (Figure 1D–F). To further observe the effect of ART on renal dysfunction induced by cisplatin, we performed H&E and PAS staining, and the results demonstrated that ART significantly decreased renal damage (Figure 1G). These findings verified that ART ameliorated renal dysfunction in Cis‐induced AKI.

### 3.2 Artesunate suppressed renal inflammation and renal necroptosis in Cis‐induced AKI

**FIGURE 2:**
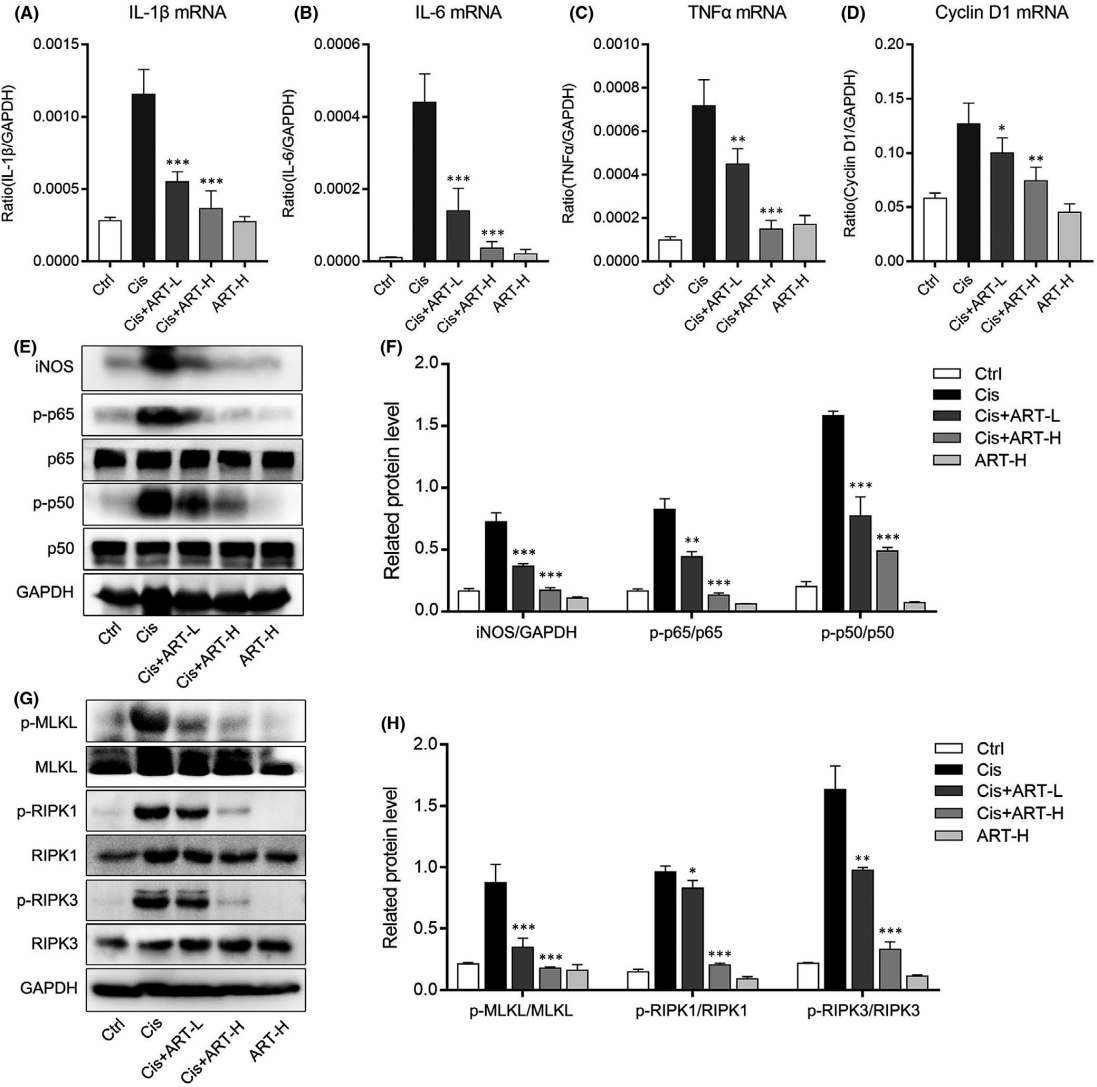
Artesunate suppressed renal inflammation and renal necroptosis in Cis‐induced AKI. (A–D) The mRNA expression of IL‐1β, IL‐6, TNF‐α and Cyclin D1 tested by real‐time PCR in kidney of each group. (E and F) The protein levels of iNOS, p‐p65 and p‐p50 detected by Western blotting in kidney of each group. (G and H) The protein levels of p‐RIPK1, p‐RIPK3 and p‐MLKL detected by Western blotting in kidney of each group. *p < 0.05, **p < 0.01, ***p < 0.001 vs. Cis group

Numerous previous studies have shown that inflammation plays an important role in acute kidney injury. To examine whether ART was able to improve the Cis‐induced inflammatory response, we employed real‐time PCR to detect the expression of inflammatory cytokines in kidney. As we expected, the mRNA levels of IL‐1β, IL‐6, TNF‐α and Cyclin D1 in kidney tissues dramatically increased with Cis stimulation, while the mRNA levels of these cytokines were remarkably reduced after ART treatment (Figure 2A–D). Meanwhile, we detected the protein level of iNOS, p‐p65 and p‐p50 in kidney of AKI. As a result, we found that ART can down‐regulate the protein levels of iNOS, and activation of p‐p65 and p‐p50 in kidney of AKI (Figure 2E,F). In addition, we performed Western blotting to detect the activation of p‐MLKL, p‐RIPK1 and p‐RIPK3 (Figure 2G,H). The results revealed that ART decreased the activation of necroptosis‐related proteins, such as p‐RIPK1, p‐RIPK3 and p‐MLKL in Cis‐induced AKI. The above data suggested that ART suppressed renal inflammation and necroptosis through inhibiting NF‐κB and RIPK1/RIPK3/MLKL pathway in Cis‐induced AKI.

### 3.3 Artesunate inhibited Mincle‐maintained M1 macrophages activation in AKI

**FIGURE 3:**
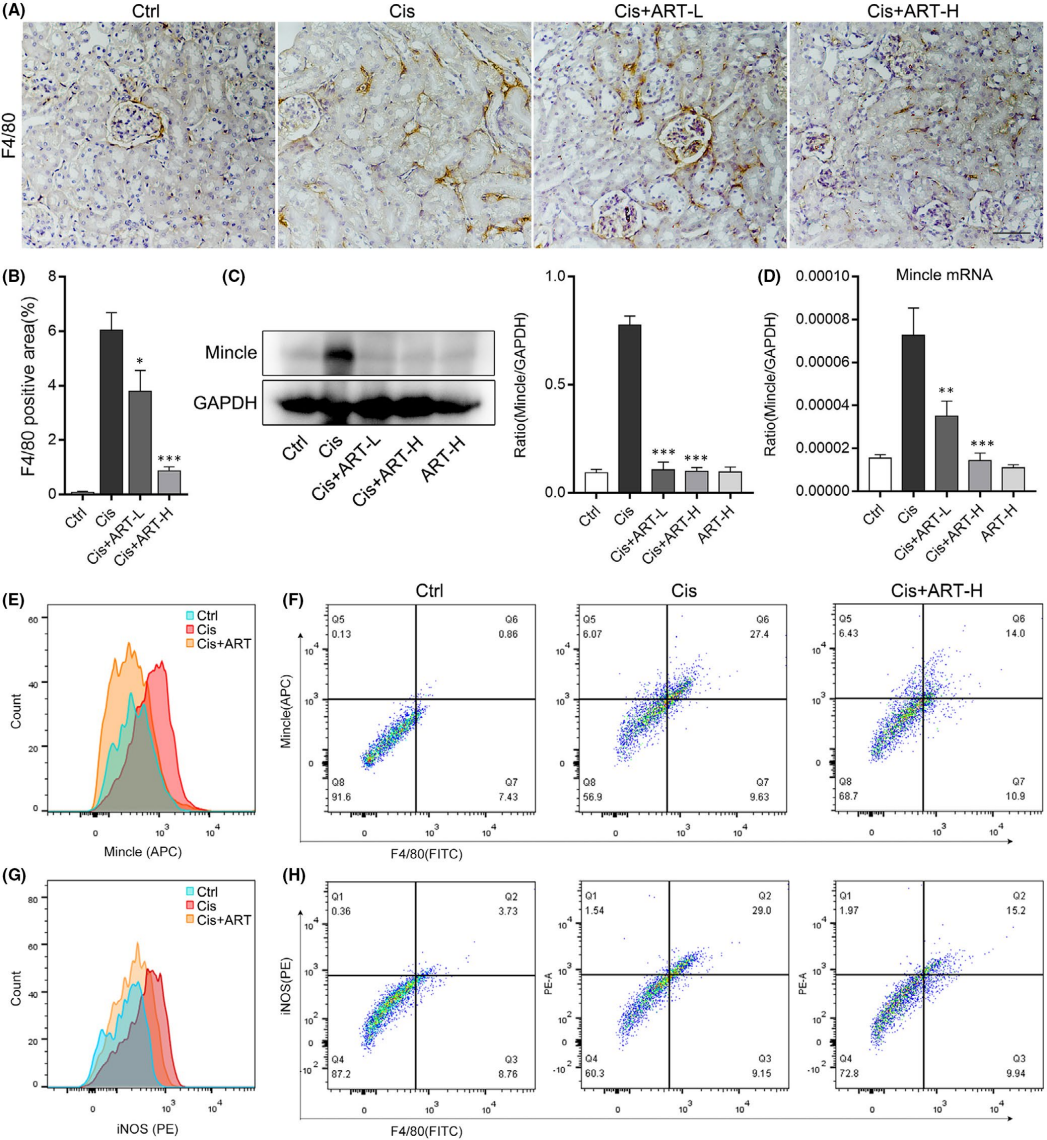
Artesunate inhibited Mincle‐maintained M1 macrophages activation in AKI. (A and B) Immunohistochemistry staining of F4/80 in kidney of AKI mice. (C) The relative protein level of Mincle detected by Western blotting normalized to GAPDH in kidney of each group. (D) ART administration effectively reduced the mRNA expression of Mincle in kidney of AKI mice. (E–H) Flow results indicated the Mincle and iNOS positive macrophage in kidney, respectively. *p < 0.05, **p < 0.01, ***p < 0.001 vs. Cis group

M1 macrophages facilitate inflammation in kidney of AKI, whereas M2 macrophages suppress inflammation. Previous studies demonstrated that Mincle plays an essential role in inflammatory response of macrophage and maintains the M1 phenotype of macrophage in kidney of AKI, so we firstly detected the expression of F4/80 and Mincle in kidney tissue of AKI. The immunohistochemical staining shows that macrophages were voluminously infiltrated in kidney of AKI. Dramatically, treatment with ART prevented the infiltration of macrophages in kidney of AKI (Figure 3A,B). In addition, the mRNA and protein levels of Mincle were significantly increased in kidney of AKI, which were decreased after ART intervention (Figure 3C,D). For another, we used flow cytometry to analyse the expression of iNOS and Mincle in macrophages. As expected, the results revealed that ART intervention can effectively down‐regulate the increased expression of Mincle in macrophages and inhibit the activation of M1 macrophages (iNOS is the marker of M1 macrophage) (Figure 3E–H). In summary, we found that ART inhibited Mincle, which maintained M1 macrophage activation in kidney of AKI.

### 3.4 Artesunate improved the morphology of LPS‐stimulated BMDM

**FIGURE 4:**
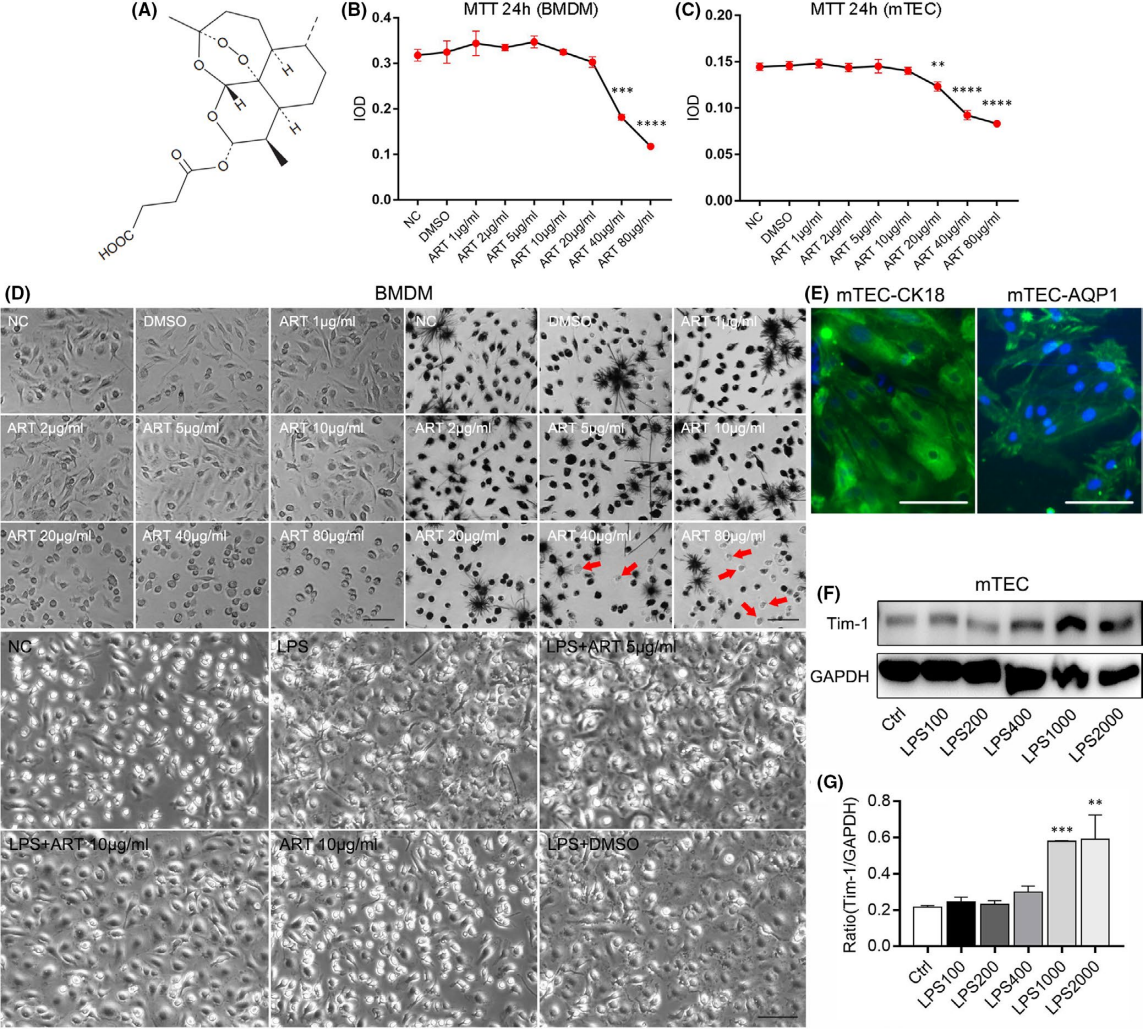
Artesunate improved the morphology of LPS‐stimulated BMDM. (A) This figure shows the chemical structure of ART. (B) The effect of ART on the survival rate of BMDM. (C) The effect of ART on the survival rate of mTEC. (D) ART reversed the morphology change of LPS‐stimulated BMDM cells. (E) Immunofluorescence staining of AQP‐1 and CK18 (Markers of renal tubule cells) in mTEC. (F and G) The relative protein level of Tim‐1 in BMDM stimulated with different concentration of LPS. **p < 0.01, ***p < 0.001, ****p < 0.0001 vs. Ctrl group

The chemical structure of ART is shown in Figure 4A. In order to observe the therapeutic effect of ART on LPS‐stimulated macrophages and mTEC, we first tested the drug toxicity of ART on the two cells through the MTT assay. The BMDM viability was reduced with ART dose above 40 μg/ml, and mTEC viability was reduced with ART dose above 20 μg/ml after 24‐h treatment (Figure 4B–D upper. Red arrows indicate dead cells). As a result, the ART concentration in subsequent experiments was decided to be 20 μg/ml or lower. Subsequently, we observed that BMDM cells became sparse and broken after LPS stimulation, and ART visibly reversed the LPS‐stimulated morphology change (Figure 4D below). Then, we substantiated the purify of mTEC by immunofluorescence staining. CK18 and AQP‐1 were markers of primary mTEC. As shown in Figure 4E, the results demonstrated that CK18 and AQP‐1 were positive in cells, indicating the isolated cells were mTEC. We next measured the protein level of Tim‐1 in kidney of AKI to determine the optional concentration of LPS to stimulate mTEC. When the concentration of LPS reaches 1000 ng/ml, the cell damage of mTEC was significantly increased; however, the LPS concentration used in stimulation of BMDM was 200 ng/ml (Figure 4F,G).

### 3.5 Artesunate inhibited inflammation by suppressing Mincle‐related signalling pathway in BMDM

**FIGURE 5:**
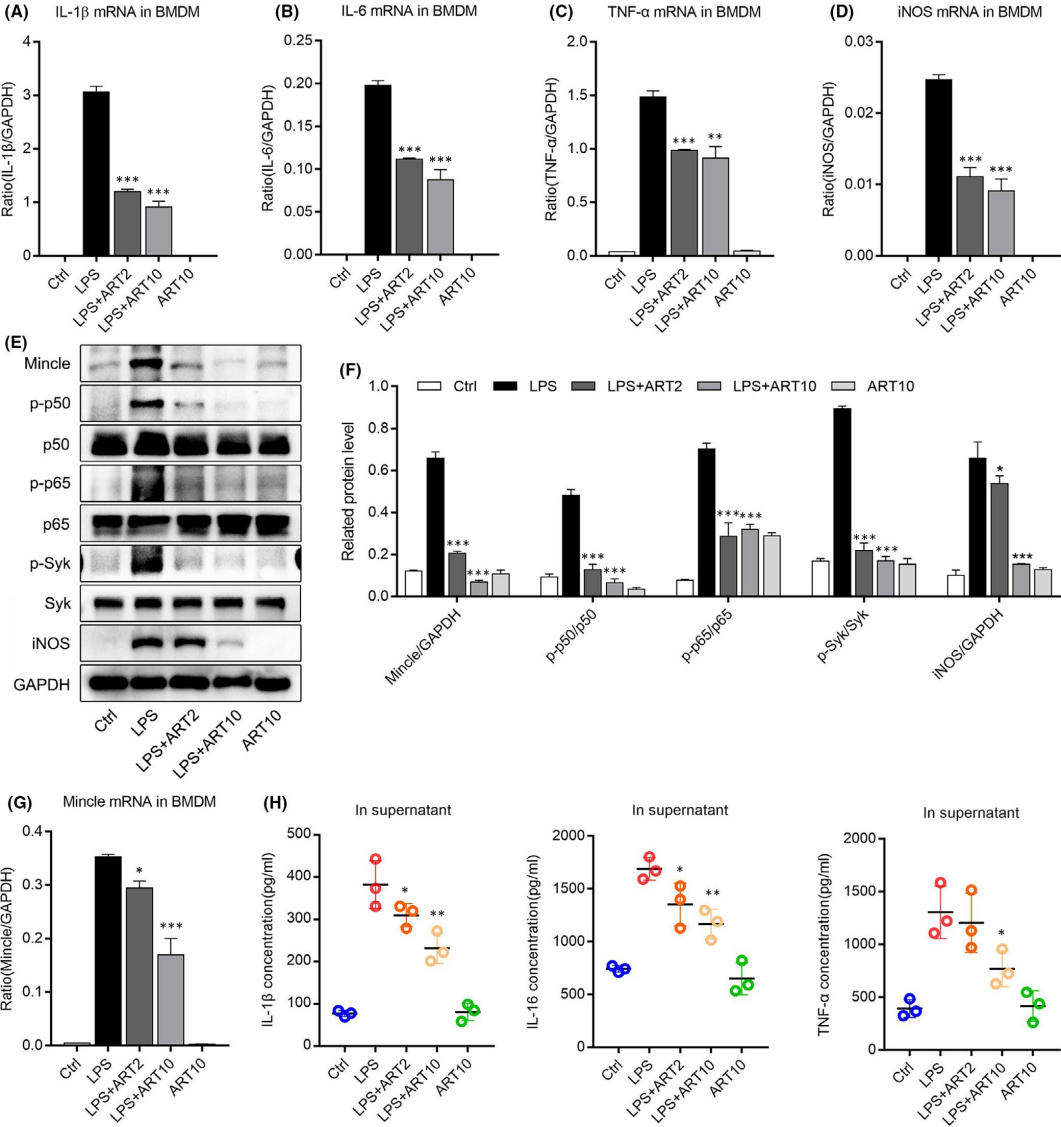
Artesunate inhibited inflammation by suppressing Mincle‐related signalling pathway in BMDM. (A–D) Real‐time PCR results showed that ART administration strongly reduced the mRNA expression of IL‐1β, IL‐6, TNF‐α and iNOS in LPS‐stimulated BMDM cells. (E and F) The relative protein levels of Mincle, p‐p65, p‐p50, p‐syk and iNOS detected by Western blotting in LPS‐stimulated BMDM cells. (G) The mRNA expression of Mincle in each group. (H) The concentration of IL‐1β, IL‐6 and TNF‐α in supernatant of each group detected by ELISA. *p < 0.05, **p < 0.01, ***p < 0.001 vs. LPS group

To identify anti‐inflammatory effect of ART on LPS‐stimulated BMDM cells, we primarily detect the mRNA expression of iNOS, TNF‐α, IL‐1β and IL‐6 in BMDM through real‐time PCR analysis. We found that the mRNA expression of iNOS and these above inflammatory cytokines was obviously reduced after ART administration in LPS‐stimulated BMDM (Figure 5A–D). Moreover, we next examined the effects of ART on suppression of Mincle‐related signalling pathway in BMDM cells. The immunoblotting results indicated that ART significantly down‐regulated the protein levels of iNOS and Mincle in LPS‐induced BMDM cells, and also down‐regulated the downstream indicators of Mincle, such as p‐p65, p‐p50 and p‐syk (Figure 5E,F). Besides, we also found that ART can visibly reduce the mRNA expression of Mincle in inflammatory BMDM (Figure 5G). Furthermore, the ELISA analysis demonstrated that the secretion of inflammatory cytokines was strongly reduced after ART treatment (Figure 5H). These findings revealed that ART inhibited inflammatory response in macrophage by suppressing Mincle‐related signalling pathway.

### 3.6 LPS‐stimulated BMDM aggravated necroptosis of mTEC

**FIGURE 6:**
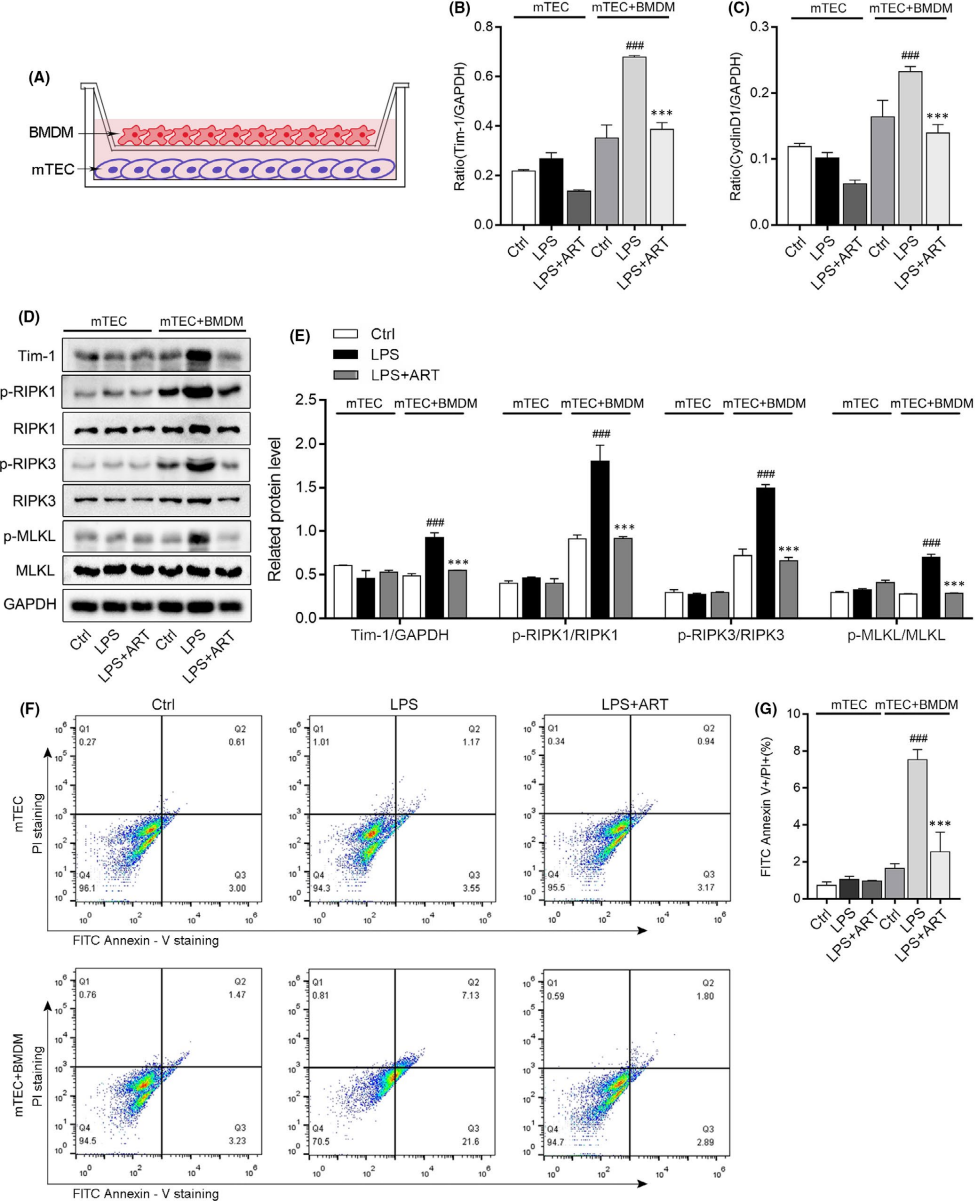
LPS‐stimulated BMDM aggravated necroptosis of mTEC. (A) The schematic diagram of the co‐culture system. (B and C) The mRNA expression of Tim‐1, and Cyclin D1 in mTEC of co‐culture system detected by real‐time PCR. (D and E) The protein levels of Tim‐1, p‐RIPK1, p‐RIPK3 and p‐MLKL in mTEC of co‐culture system detected by Western blotting. (F and G) The results of Annexin‐PI staining. ^###^ *p *< 0.001 vs. LPS in mTEC group. ***p < 0.001 vs. LPS in mTEC + BMDM group

We set up a co‐culture system to observe the effect of LPS‐stimulated BMDM on cellular damage and necroptosis to mTEC (Figure 6A). The BMDM cells were cultured in an upper transwell chamber and mTEC were cultured in a well of 6‐well plate. The results of real‐time PCR showed that the mRNA levels of Tim‐1 and cyclin D1 in mTEC were significantly increased when co‐cultured with BMDM than those groups without BMDM under stimulation of 200 ng/ml LPS (Figure 6B,C). Notably, ART can reduce the protein levels of Tim‐1 and activation of p‐RIPK1, p‐RIPK3 and p‐MLKL in mTEC co‐cultured with or without BMDM (Figure 6D,E). At last, we confirmed that ART effectively alleviated LPS‐induced necroptosis through Annexin V staining (Figure 6F,G). In brief, in an inflammatory environment (stimulation of BMDM with 200 ng/ml LPS), the inflammatory response of macrophage can significantly aggravate the damage and necroptosis of renal tubular cells, but ART can inhibit the inflammatory cross‐talk between BMDM and mTEC.

### 3.7 Artesunate improved inflammation and necroptosis of mTEC by inhibiting Mincle in BMDM

**FIGURE 7:**
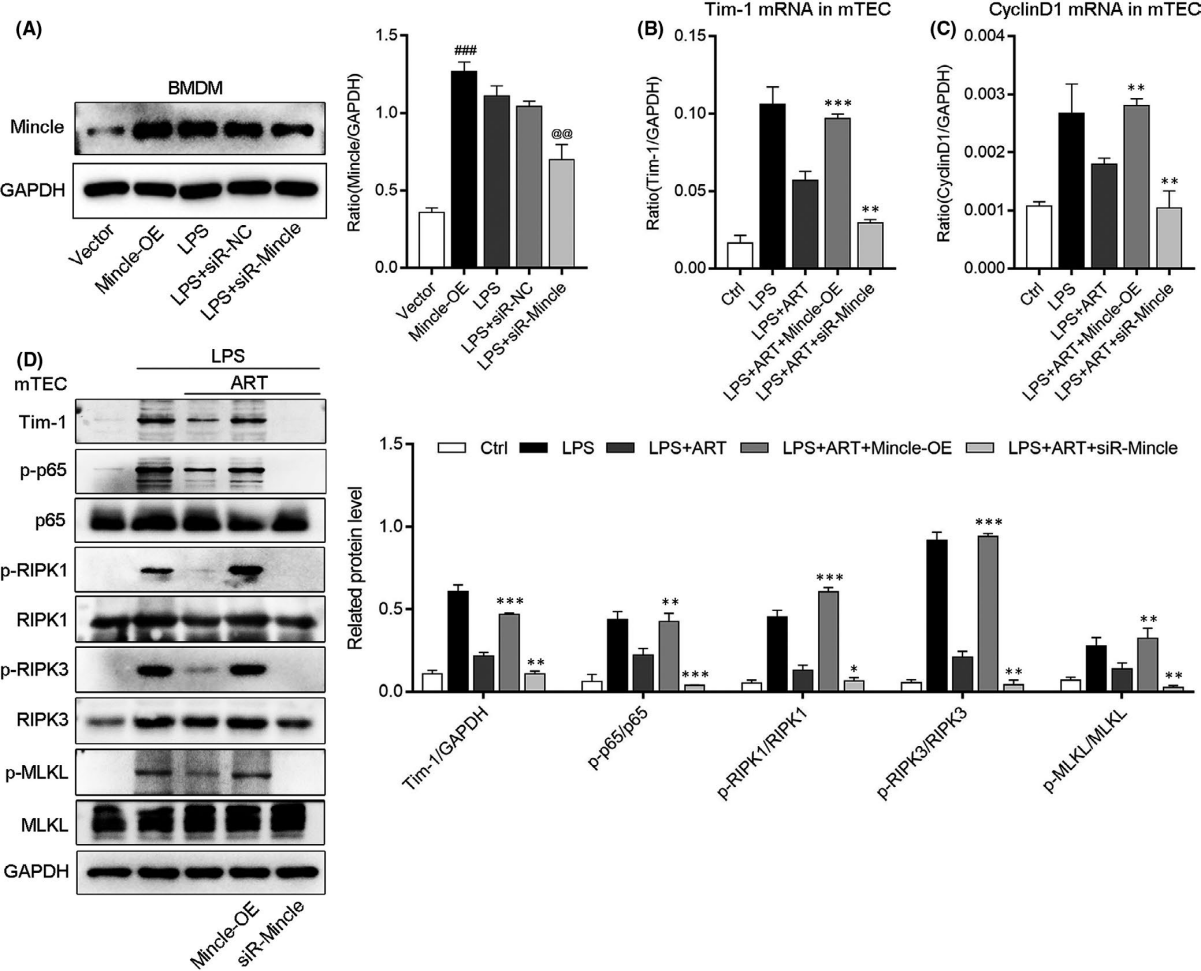
Artesunate improved inflammation and necroptosis of mTEC by inhibiting Mincle in BMDM. (A) Western blot was used to detect the protein level of Mincle in macrophage after the transfection of siRNA‐Mincle and Mincle overexpression plasmid DNA. ^###^ *p*<0.001 vs. vector group. ^@@^ *p*<0.01 vs. LPS group. (B and C) The mRNA expression of Tim‐1 and Cyclin D1 in BMDM‐co‐cultured mTEC detected by real‐time PCR. (D) The protein levels of Tim‐1, p‐p65, p‐RIPK1, p‐RIPK3 and p‐MLKL in BMDM‐co‐cultured mTEC detected by Western blotting. *p < 0.05, **p < 0.01, ***p < 0.001 vs. LPS + ART group

To further investigate the mechanism of ART on LPS‐stimulated BMDM and mTEC in AKI, we knock down and overexpress Mincle in BMDM by siRNA and plasmid transfection. The Western blot results demonstrated that the protein level of Mincle was upregulated in plasmid transfection group (OE‐Mincle) and down‐regulated in siRNA group (siRNA‐Mincle) in BMDM (Figure 7A). Therefore, we co‐cultured the transfected BMDM with mTEC to examine the potential effects of ART. We found that the mRNA expression of Tim‐1, cyclin D1 and protein levels of p‐p65 and necroptosis‐related proteins in mTEC were activated in Mincle‐OE group and suppressed in siRNA‐Mincle group, respectively (Figure 7B–D). Notably, the overexpression of Mincle in LPS‐induced BMDM reversed the inflammatory and necroptosis inhibitory effects of ART on mTEC, suggesting that ART improved inflammation and necroptosis of mTEC by inhibiting Mincle in BMDM.

## 4 DISCUSSION

In the present study, we explored the potentially protective effects of artesunate on cisplatin‐induced AKI mouse as well as LPS‐stimulated BMDM and mTEC. All the findings indicated that artesunate improved renal function and inhibited inflammation and necroptosis of kidney in cis‐induced AKI in vivo, while suppressed macrophage‐mediated inflammatory response and necroptosis to mTEC in vitro according to a co‐culture system. Notably, these results showed that artesunate effectively alleviates inflammation and necroptosis of mTEC by down‐regulating Mincle on macrophage may be the essential mechanism of improving AKI by artesunate. In summary, artesunate relieves cis‐induced AKI through inhibiting Mincle‐mediated macrophagic inflammatory response.

In addition to its significant anti‐malarial effect, artesunate also has other antiviral effects, such as anti‐herpes virus^30^ and hepatitis B virus.^31^ In addition, artesunate also has an antiparasitic effect, including anti‐trypanosoma,^32^ toxoplasma gondii^33^ and schistosoma.^34^ In recent years, the anti‐inflammatory effects of artesunate have attracted more and more attention, including anti‐autoimmune diseases (rheumatoid arthritis,^35^ inflammatory bowel disease^36^ and systemic lupus erythematosus^37^), allergic inflammation,^38^ septic inflammation^39^ and Alzheimer's disease.^40^ However, there is no report on the role of artesunate in inflammatory disease of AKI. Therefore, we hope to verify whether artesunate has a protective effect on kidney of AKI and its potential mechanism. In view of the immune effect of artesunate, we hope to start with the inhibition of artesunate on the inflammation of infiltrated macrophage in kidney of AKI, and explore its possible renoprotective mechanism.

It is well known that inflammation is the dominating pathogenesis of cisplatin‐induced AKI.^41^ In the kidney of AKI, macrophage can trigger renal inflammation and recruit other immune cells, eventually causing the release of a large number of inflammatory cytokines, resulting serious kidney damage.^42^ Notably, Mincle has been confirmed by studies to be a key factor for maintaining M1 type macrophage, and it plays an important role in kidney inflammation of AKI.^20^, ^43^, ^44^ Our previous research also reported that Quercetin protects against cis‐stimulated AKI by inhibiting Mincle/Syk/NF‐κB signalling maintained macrophage inflammation.^45^ However, it is not clear how Mincle slows down the damage of kidney in AKI. In this study, we explored the potential mechanism of inhibiting Mincle to improve AKI and found that LPS‐stimulated macrophage can aggravate the necroptosis of co‐cultured renal tubular cells, and this concentration of LPS did not stimulate the inflammatory response of renal tubules alone without macrophage. After knocking down the expression of Mincle in macrophage by siRNA, we found that the damage of co‐cultured renal tubular cells was reduced under the stimulation of LPS, and artesunate could also improve the inflammation and necroptosis of mTEC by inhibiting the expression of Mincle in macrophage, indicating that Mincle on macrophage is a key target for artesunate to improve AKI. Notably, the results of in vitro study showed that the ability of artesunate to inhibit the protein level of Mincle was significantly higher than that of the expression of Mincle mRNA, which may be because of the further inhibition of the post‐transcriptional translation of Mincle by artesunate, and the specific effect and mechanism need to be further studied in subsequent experiments. Moreover, our results showed that although artesunate and the positive control curcumin have equal anti‐inflammatory effects, artesunate exhibited a significant inhibitory effect on macrophage inflammatory response, which proves the immunomodulatory effect of ART previously reported by other researchers, that is beneficial to develop other properties of artesunate besides antibacterial.

In addition, necroptosis is a form of programmed necrosis and is striked by activation of RIPK1/RIPK3/MLKL signalling pathway and plays a significant role in AKI.^46^, ^47^ When the death signal is transmitted to RIPK1, RIPK1 will bind to RIPK3 and phosphorylate MLKL, ultimately leading to cell necroptosis.^48^, ^49^ In this present study, the results showed that artesunate remarkably reduced the mRNA and protein levels of RIPK1, RIPK3 and cyclin D1 as well as phosphorylation of MLKL in cis‐induced AKI and LPS‐stimulated mTEC, indicating that artesunate significantly inhibited necroptosis. Macrophage and renal tubular cells are the main cells involved in the pathological process of kidney in AKI. By establishing a cell co‐culture system, the results of real‐time PCR and Western blot indicated that the mRNA levels of Tim‐1, cyclin D1, RIPK1 and RIPK3 in mTEC co‐cultured with LPS‐stimulated BMDM cells were clearly elevated than those groups without BMDM cells. In brief, LPS‐stimulated BMDM cells aggravated necroptosis of mTEC. Moreover, the results also proved that Mincle on macrophages can not only aggravate the inflammation, but also up‐regulate the necroptosis of renal tubular cells. Notably, artesunate can significantly inhibit macrophage‐induced necroptosis of renal tubular. However, after overexpression of Mincle, the inhibitory effect of artesunate on necroptosis in mTEC disappeared, indicating that artesunate reduces renal tubular cell necroptosis by suppressing Mincle on macrophage.

In summary, this study found that artesunate can significantly reduce renal damage and necroptosis and improve renal dysfunction and inflammation in kidney of AKI mouse model, and its mechanism is mainly related to inhibit macrophagic Mincle‐mediated necroptosis and inflammation to tubular epithelial cell. In short, artesunate inhibits the activation of M1 macrophages and the RIPK1/RIPK3/MLKL signalling pathway by down‐regulating the expression of Mincle, thus reducing the inflammatory response and necroptosis, and further improving the renal injury of AKI. These findings provide a new theoretical direction for AKI treatment.

## CONFLICTS OF INTEREST

The authors declare that there are no conflicts of interest.

## AUTHOR CONTRIBUTIONS

**Xian‐ying Lei:** Conceptualization (equal); Investigation (lead); Methodology (equal); Writing‐original draft (lead). **Rui‐zhi Tan:** Methodology (equal); Project administration (equal); Software (equal); Writing‐original draft (equal); Writing‐review & editing (equal). **Jian Jia:** Formal analysis (equal); Software (equal); Visualization (equal); Writing‐original draft (equal). **Song‐lin Wu:** Investigation (equal); Methodology (equal); Software (equal). **Cheng‐li Wen:** Methodology (equal); Software (equal); Visualization (equal). **Xiao Lin:** Software (equal); Writing‐original draft (equal); Writing‐review & editing (equal). **Huan Wang:** Investigation (equal); Visualization (equal). **Zhang‐jing Shi:** Data curation (equal); Validation (equal). **Bo Li:** Writing‐review & editing (equal). **Yan Kang:** Supervision (equal); Writing‐review & editing (equal). **Li Wang:** Conceptualization (lead); Funding acquisition (lead); Resources (equal); Supervision (equal); Writing‐review & editing (equal).

## Data Availability

The data that support the findings of this study are openly available.
